# A case series of a mother and two daughters with a *GLI2* gene deletion demonstrating variable expressivity and incomplete penetrance

**DOI:** 10.1002/ccr3.3085

**Published:** 2020-08-30

**Authors:** Cameron Elward, Janet Berg, John M. Oberlin, Luis Rohena

**Affiliations:** ^1^ Division of Genetics Department of Pediatrics Brooke Army Medical Center Joint Base San Antonio Texas USA; ^2^ Division of Endocrinology Department of Pediatrics Brooke Army Medical Center Joint Base San Antonio Texas USA; ^3^ Department of Pediatrics Long School of Medicine University of Texas Health San Antonio Texas USA

**Keywords:** Culler Jones syndrome, GLI2, holoprosencephaly, hypopituitarism

## Abstract

This case series and review of the literature support that patients with pathogenic variants of the GLI2 gene demonstrate an autosomal dominant inheritance pattern, variable expressivity, and incomplete penetrance.

## Graphical abstract

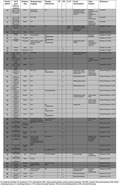

## 1 INTRODUCTION

In humans, mutations in *GLI2* gene, a mediator of the Sonic Hedgehog (SHH) pathway, can result in holoprosencephaly (HPE), which is the failure of midline cleavage of the forebrain during embryonal development. Clinical manifestations can range from malformed or absent cerebral tissue to hypotelorism, or narrowly spaced eyes, to cyclopia. In murine models, SHH has been shown to be expressed in the ventral diencephalon as well as the midline structures such as the notochord and floor plate of the notochord, identifying its role in pituitary and limb development, respectively.^1^ Thus, the *GLI2* gene has been implicated to be involved in forebrain, pituitary, and limb development.^2^, ^3^, ^8^, ^9^, ^11^

In this series, we present a family, two daughters and a mother, with an identical *GLI2* gene mutation. Sisters 1 and 2 present with the classic dyad of findings in a patient with Culler Jones syndrome of growth hormone (GH) deficiency and postaxial polydactyly, and their mother also demonstrated postaxial polydactyly at birth but otherwise has an unremarkable phenotype.

We also reviewed the literature for previously described families of two members or more that have a pathogenic *GLI2* gene mutation. To our knowledge, there are only seven previously described families of two or more members with the *GLI*2 gene mutation or deletion, collectively they total thirty‐one patients. The content of this manuscript is not considered research at our institution and instead falls in the realm of routine clinical care. Informed consent for publication was obtained from the parents.

## 2 CLINICAL SERIES

Sister 1 is a 12‐year‐old girl born to nonconsanguineous, German/Eastern European and Polish/Irish parents. There is also Ashkenazi Jewish ancestry in the family. She was referred to genetics for evaluation of the *GLI2* gene deletion, which was also later confirmed on testing in her younger sister and mother, in the setting of an isolated GH deficiency and ectopic posterior pituitary. The patient was born by induced vaginal delivery at 40 + 3 weeks gestation, birth weight (BW) of 3.4 kg (41st centile). Mother was positive for Group B Streptococcus (GBS), which was adequately treated with two doses of antibiotics. Her newborn screen was normal. She was transferred to the neonatal intensive care unit (NICU) for observation for jaundice and hypoglycemia and removal of right hand postaxial polydactyly on the first day of life, and she was discharged at 7 days. She was identified to have failure to thrive at around 6 months of life despite adequate caloric intake; laboratory work‐up showed low IGF‐1, and follow‐up laboratory studies confirmed a diagnosis of GH deficiency and was subsequently placed on daily subcutaneous somatotropin injections. At around 1 year of life, the patient had gastroparesis demonstrated by gastric emptying study and reflux demonstrated by esophagoduodenoscopy so she was started on lansoprazole, metoclopramide, and erythromycin. Due to persistent difficulty in obtaining adequate oral calories, a gastric tube was placed at 19 months and then removed at 4 years when oral intake became adequate. Array‐based comparative genomic hybridization (aCGH) was performed and showed a small 90kb interstitial deletion of chromosome 2 including a portion of the *GLI2* gene. Brain magnetic resonance imaging (MRI) without contrast showed an ectopic posterior pituitary gland and Chiari I malformation. The patient's examination was significant for hypotelorism, bilateral epicanthal folds, a flat nasal bridge, a bulbous nasal tip, and bilateral clinodactyly of digits 2, 3, 4, and 5. The patient at 11 years old had a weight of 41.6 kg (65th centile) and length of 141.7 cm (31st centile) and was tracking appropriately on both curves. There were no concerns with development or cognition.

**FIGURE 1:**
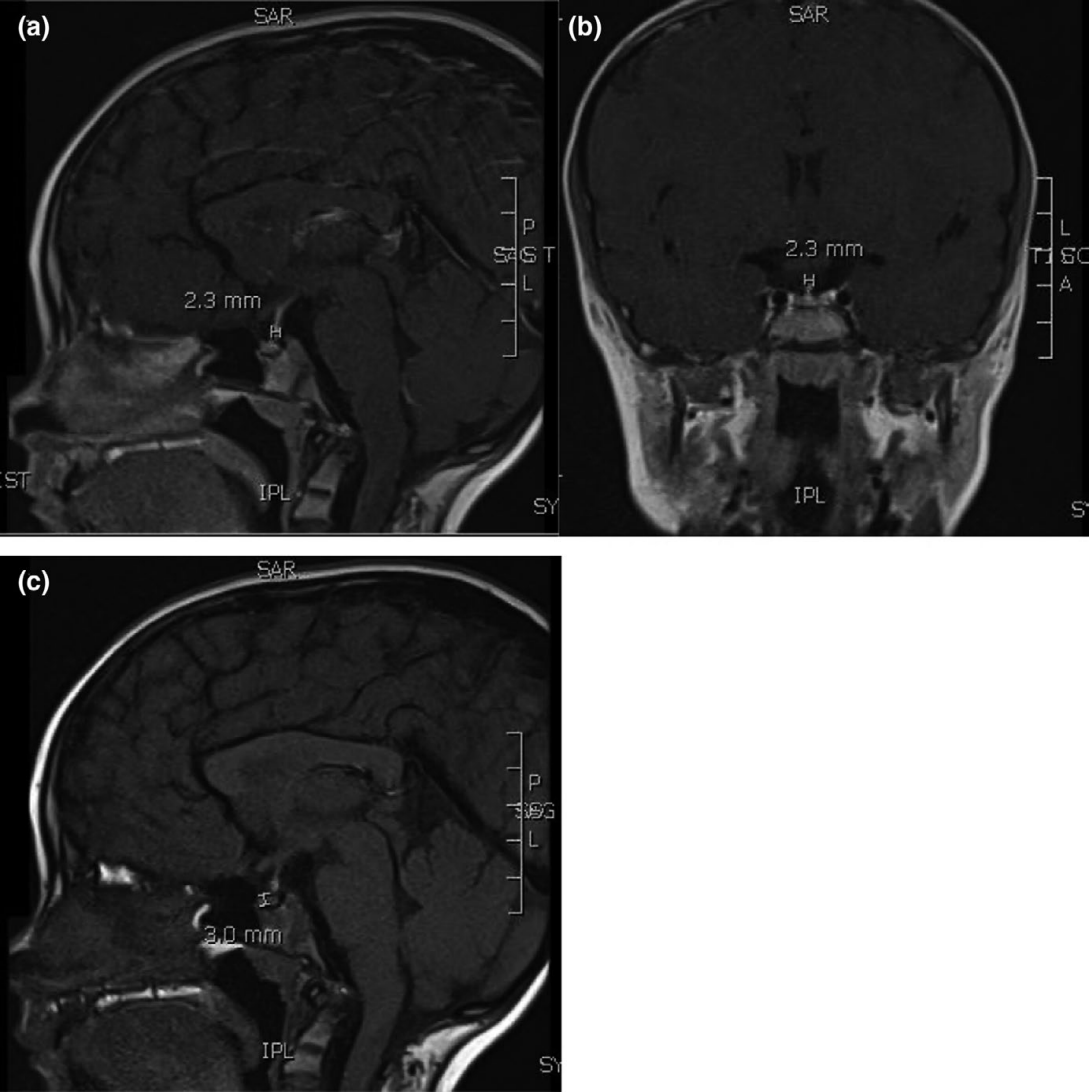
Magnetic resonance imaging of Sister 2. MRI of brain and sella was performed with and without gadolinium‐based intravenous contrast. Coronal and sagittal T1‐weighted images show ectopic posterior pituitary (EPP) and anterior pituitary hypoplasia. (A and B) There is a focal T1 hyperintense thickening of the infundibulum measuring 2.3 mm with a normal posterior pituitary bright spot not seen within the sella most consistent with an EPP. (C) The anterior pituitary gland is small measuring 3 mm in height

Sister 2 is an 8‐year‐old girl born to the same parents. She was referred to genetics for a more thorough work‐up for her short stature, history of failure to thrive, and a sister known to have a previous genetic work‐up which revealed a *GLI2* gene deletion. She was confirmed to have the same deletion on chromosome 2 as her sister. Of note, her father has a stature of 69 inches, and her mother a stature of 61.5 inches. The patient was born by spontaneous vaginal delivery at 40 + 0 weeks gestation, BW of 3.2 kg (21st centile). Her natal stay was significant for transfer to the NICU for observation for jaundice and hypoglycemia and removal of left hand postaxial polydactyly on the first day of life. She was discharged home after 48 hours. Around 1 year of life, chromosomal microarray identified a heterozygous deletion for the *GLI2* gene so an arginine/glucagon stimulation test for GH deficiency was performed and showed a GH peak >10 ng/mL (peak: 11.8 ng/mL) and a cortisol peak >18 mcg/dL (peak: 33.1 mcg/dL), which ruled out GH deficiency and adrenal insufficiency, respectively, at that time. Given persistent short stature, patient was referred to genetics at 5 years of life, and repeat whole‐genome aCGH and single nucleotide polymorphism analysis was performed, and the patient was found to have an interstitial deletion of at least 90 kb within cytogenetic band 2q14.2 involving the *GLI2* gene. The patient's examination was significant for height and weight less than the 3rd centile and bilateral clinodactyly of digits 4 and 5. Around 7 years of life, a brain MRI with and without contrast was performed, which showed a hypoplastic anterior pituitary and ectopic posterior pituitary Figure 1. The patient's growth velocity had averaged about 5.2 cm/year in the last year, and she remained below the 3rd centile for height and weight. Given the less than expected growth in the setting of *GLI2* deletion and imaging findings, the patient was started on a trial of recombinant human GH dosing given the high likelihood of relative GH deficiency.

The mother has a history of postaxial polydactyly but is otherwise phenotypically normal. Whole‐genome aCGH revealed identical, heterozygous, interstitial 90 kb deletion within cytogenetic band 2q14.2 involving exons 3‐13 of the *GLI2* gene (ISCN: arr[GRCh37] 2q14.2 [121696090_121786360] x1) identical to that found in her daughters, which is classified as a pathogenic variant.

## 3 GENETIC TESTING

Whole‐genome array‐based comparative genomic hybridization (aCGH) and genotype analyses were performed on a custom‐designed oligonucleotide microarray (GenomeDx v5) by GeneDx. The array design is based on human genome build GRCh37/UCSC hg19, and results are reported according to ISCN guidelines. The array contains approximately 11 8000 probes that provide copy number data and 6 6000 probes that generate genotype information through analysis of SNPs. Reported boundaries correspond to deviating probes, which are dependent on array design and have the inherent limitation of not reflecting exact aberration breakpoints. The array detects copy number changes of >200 kb, on average, across the entire unique sequence of the human genome and between 500 bp and 15 kb in more than 200 targeted regions for testing performed on blood samples, which was the specimen type for the patients in this report.

## 4 DISCUSSION

After literature review identified previously published cases of families with a *GLI2* gene mutation, Table 1 was created to compare the characteristics with that of the patients in this case series. Some of the most commonly reported characteristics, including anterior pituitary lobe hypoplasia, ectopic posterior pituitary lobe identified on MRI, the resulting pituitary deficiencies, postaxial polydactyly, and facial dysmorphisms, most commonly cleft lip and palate, were given their own column in order to facilitate comparing and contrasting the different families' characteristics. True holoprosencephaly was only identified in 2/37 patients and was the rarest characteristic identified on this review, although not all patients had imaging since it was obtained when clinically indicated in most cases Table 2.^4^, ^5^, ^6^, ^7^, ^10^ Over half (20/37) of the *GLI2* gene mutation patients in this study were identified to have polydactyly, making it the most common characteristic.^4^, ^5^, ^6^, ^7^, ^10^

**TABLE 1:** Comparison of features in seven prior reported families with *GLI2* gene deletion or mutations

Family/patient	GLI2 gene deletion/mutation	Relation‐ship	Brain/pituitary imaging	Pituitary deficiencies	ID	PD	CL/P	Facial dysmorphism	Other features	References
1a	2q14.2(121696090_121786360) deletion	Sister 1	EPP, Chiari I malformation	GH	‐	+	‐	Hypotelorism, bilateral epicanthal folds, flat nasal bridge, a bulbous nasal tip	Moderate hypermobility and bilateral clinodactyly of 2/3/4/5	Our patient
1b	2q14.2(121696090_121786360) deletion	Sister 2	EPP, APH	‐	‐	+	‐	‐	Severe hypermobility	Our patient
1c	2q14.2(121696090_121786360) deletion	Mother	N/A	‐	‐	+	‐	‐	Madelung deformity and hypermobility	Our patient
2a	IVS5 + 1G > A mutation	Son	Normal	GH	+	‐	Pseudo‐median CL	Hypotelorism; single nares; extreme midface hypoplasia; and microcephaly	‐	10
2b	IVS5 + 1G > A mutation	Father	N/A	‐	‐	‐	‐	‐	‐	10
3a	2274del1	Sister	Absent pituitary	Pan ‐hypopituitarism	‐	+	+	‐	Optic nerve hypoplasia	10
3b	N/A (deceased)	Twin brother 1	N/A	Pan ‐hypopituitarism	‐	‐	‐	‐	‐	10
3c	2274del1	Twin brother 2	Absent pituitary, partial agenesis of corpus callosum	Pan ‐hypopituitarism	‐	‐	+	Hypotelorism and flat midface	Abnormal configuration of lower midline structures	10
3d	2274del1	Father	N/A	‐	‐	+	‐	‐	‐	10
3e	2274del1	Paternal aunt	N/A	‐	‐	+	‐	‐	‐	10
4a	2q14.2(120483663_121811865) deletion	Sister 1	EPP, APH	+	‐	‐	+	Mild midface hypoplasia and flat nasal tip	‐	6
4b	2q14.2(120483663_121811865) deletion	Sister 2	N/A	‐	‐	‐	‐	‐	Unilateral conductive hearing loss	6
4c	2q14.2(120483663_121811865) deletion	Mother	N/A	‐	‐	‐	‐	‐	‐	6
5a	c.3676C > T (p.Arg1226) mutation	Son	EPP, APH	Pan ‐hypopituitarism		+	‐	Hypotelorism, ribbed palatum durum, single median incisor	Midurethral stenosis with urethral valves, cryptorchidism	7
5b	c.3676C > T (p.Arg1226) mutation	Father	N/A	‐	‐	+	‐	Ribbed palatum durum	‐	7
5c	c.3676C > T (p.Arg1226) mutation	Sister 1	N/A	‐	‐	+	‐	Ribbed palatum durum	‐	7
5d	c.3676C > T (p.Arg1226) mutation	Sister 2	N/A	‐	‐	+	‐	Ribbed palatum durum	‐	7
5e	c.3676C > T (p.Arg1226) mutation	Sister 3	N/A	Pan‐hypopituitarism	‐	‐	‐	‐	‐	7
5f	c.3676C > T (p.Arg1226) mutation	Brother	N/A	‐	‐	‐	‐	Ribbed palatum durum, narrowed meatus ear	Cardiac septum defect	7
5g	c.3676C > T (p.Arg1226) mutation	Sister 4	N/A	‐	‐	‐	‐	‐	Cardiac septum defect, urethral meatus stenosis	7
5h	c.3676C > T (p.Arg1226) mutation	Niece 1	EPP	Pan ‐hypopituitarism	‐	‐	‐	Hypotelorism	‐	7
5i	c.3676C > T (p.Arg1226) mutation	Niece 2	EPP	Pan ‐hypopituitarism	‐	‐	‐	‐	‐	7
5j	c.3676C > T (p.Arg1226) mutation	Niece 3	EPP	Pan ‐hypopituitarism	‐	‐	‐	‐	‐	7
6a	c.2362_2368del p.L788fsX794	Daughter	Diminished brain size, asymmetry of cerebral hemispheres, EPP, APH	PRL, GH, FSH, LH, ACTH, TSH		+			Vesicoureteral reflux	5
6b	c.2362_2368del p.L788fsX794	Maternal grandmother	N/A	‐	‐	+	‐	‐	‐	5
6c	c.2362_2368del p.L788fsX794	Mother	N/A	‐	‐	+	‐	‐	‐	5
6d	c.2362_2368del p.L788fsX794	Maternal uncle 1	EPP, APH	GH	‐	+	‐	‐	‐	5
6e	c.2362_2368del p.L788fsX794	Maternal uncle 2	EPP, APH, thin pituitary stalk at infundibulum, ventricular dilation, and several sequelae of head trauma	GH, FSH, LH	‐	+	‐	‐	Suffered severe head trauma 10 y prior to evaluation	5
6f	c.2362_2368del p.L788fsX794	Maternal uncle 3	N/A	‐	‐	+	‐	‐	‐	5
6g	c.2362_2368del p.L788fsX794	Cousin	N/A	GH, FSH, LH, TSH	‐	+	‐	‐	‐	5
7a	c.2081_2084del p.L694fsX722	Son	EPP, APH	GH, ACTH (partial)	‐	‐	+	Flat nasal bridge	Unilateral cryptorchidism	5
7b	c.2081_2084del p.L694fsX722	Father	Normal	‐	‐	‐	‐	‐	‐	5
8a	c.1138 G > T p.E380X	Daughter	APH, absence of posterior pituitary	GH, ACTH, TSH, ADH	‐	‐	‐	‐	‐	5
8b	c.1138 G > T p.E380X	Mother	N/A	‐	‐	‐	‐	‐	‐	5
9a	c.1957_2A > C mutation	Son	APH, EPP, absent pituitary stalk	GH	‐	+	‐	Midface hypoplasia, high palatal arch	Micropenis, bilateral cryptorchidism	4
9b	c.1957_2A > C mutation	Brother	N/A	‐	‐	+	‐	Mild facial hypoplasia	‐	4
9c	c.1957_2A > C mutation	Father	N/A	‐	‐	+	‐	Mild facial hypoplasia	‐	4

Abbreviations: ACTH, adrenocorticotropic hormone; ADH, antidiuretic hormone; APH, anterior pituitary hypoplasia; CL/P, cleft lip/palate; EPP, ectopic posterior pituitary; FSH, follicle stimulating hormone; GH, growth hormone; ID, intellectual disability; LH, luteinizing hormone; PD, polydactyly; PRL, prolactin; TSH, thyroid stimulating hormone.

**TABLE 2:** Ratio of common features observed in *GLI2* gene mutation or deletion in study population

Affected Region	Feature	Ratio (# with feature/total patients)
Brain/Pituitary Anomalies		
	True holoprosencephaly	2/37
	Ectopic posterior pituitary	12/37
	Anterior pituitary hypoplasia	9/37
	Panhypopituitarism	8/37
	Isolated growth hormone deficiency	3/37
Facial dysmorphisms		
	Cleft lip and palate	5/37
	Hypotelorism	5/37
Limb involvement		
	Polydactyly	20/37

The proband and her younger sister both presented with the classic dyad of Culler Jones, the mild presentation of *GLI2* gene mutation characterized by GH deficiency and postaxial polydactyly, while their mother had postaxial polydactyly and otherwise asymptomatic for pituitary deficiencies. Our case exemplified the variations in presentation among family members with the same genotype. On examination of previously published families expressing an identical *GLI2* mutation in the literature Table 1, the expression of the gene mutation varies widely among family members from normal phenotype to pituitary, facial, and limb abnormalities, confirming the pattern identified with the cases presented in this case series.^4^, ^5^, ^6^, ^7^, ^10^ This case series and literature review support that patients with pathogenic variants of the *GLI2* gene demonstrate autosomal dominant inheritance, variable expressivity, and incomplete penetrance. However, many of the parents in the literature did not receive brain imaging since it was not clinically indicated so it is possible that the findings noted are under‐representative of the severity of the phenotypes.^4^, ^5^, ^6^, ^7^, ^10^ We recommend brain MRI imaging in such patients in order to better characterize pituitary deficiencies and associated hormonal deficiencies and to better inform possible clinical intervention.

## CONFLICT OF INTEREST

None of the authors have any conflicts of interest to report.

## AUTHOR CONTRIBUTIONS

CE: gathered patient information, conducted the literature review, and synthesized the clinical information to write this report. JB: conducted detailed genetic patient histories and clarified information during the editing process. JM.O: provided patient updates throughout the writing and revising of this manuscript. LR: was the principal mentor for the project and revised several manuscripts.
